# Activation of the kynurenine pathway is associated with poor outcome in Pneumocystis pneumonia patients infected with HIV: results of 2 months cohort study

**DOI:** 10.1186/s12879-019-3851-4

**Published:** 2019-03-04

**Authors:** Mengyan Wang, Xiaotian Dong, Ying Huang, Junwei Su, Xiahong Dai, Yongzheng Guo, Caiqin Hu, Qihui Zhou, Biao Zhu

**Affiliations:** The Department of Infectious Diseases, State Key Laboratory for Diagnosis and Treatment of Infectious Diseases, Collaborative Innovation Center for Diagnosis and Treatment of Infectious Diseases, the First Affiliated Hospital, College of Medicine, Zhejiang University, Hangzhou, China

**Keywords:** Pneumocystis pneumonia, Indoleamine 2, 3-dioxygenase, Diagnostic, Mortality

## Abstract

**Background:**

Indoleamine 2, 3-dioxygenase (IDO) is a key enzyme in the degradation of tryptophan (Trp) to kynurenine (Kyn). We measured IDO activity as the Kyn to Trp ratio, and investigated whether IDO could be used to assess prognosis of acquired immune deficiency Sydrome (AIDS) patients with pneumocystis pneumonia (PCP).

**Methods:**

The Kyn and Trp concentration were measured by UPLC-MS/MS in plasma samples. A total of 49 AIDS-PCP patients were included in the analysis. Clinical characteristics and Kyn/Trp ratio were compared between survivors and non-survivors.

**Results:**

Kyn/Trp ratio was significantly lower after anti-PCP treatment in AIDS patients with PCP (*P* < 0.0001). Plasma Kyn/Trp ratio was higher in patients with PaO2/FiO2 ≤ 300 mmHg than in those with PaO2/FiO2 > 300 mmHg (*P* = 0.007). Kyn/Trp ratio, D-dimer and CRP showed much higher AUC for predicting death of AIDS-PCP patients. Kyn/Trp ratio was useful for predicting the mortality of AIDS-PCP due to a significantly higher Kyn/Trp ratio in the non-survivors (*P* = 0.002). And the high Kyn/Trp ratio group had higher mortality rate than low Kyn/Trp group (32.1% vs. 9.1%, respectively, *p* = 0.024).

**Conclusion:**

Activation of the kynurenine pathway is associated with the severity and fatal outcomes of AIDS patients with pneumocystis pneumonia.

## Background

Pneumocystis pneumonia (PCP) is a common opportunistic infection, and a significant health concern for HIV-infected patients [1]. After a combination of antiretroviral therapy (ART) and PCP prophylaxis, the PCP incidence among HIV-infected individuals has significantly decreased worldwide [2]. However, PCP continues to occur in HIV-infected patients who are not aware of their HIV infection status, and present with late-stage disease [3].

Some studies have found C-reactive protein (CRP) and lactate dehydrogenase (LDH) can be utilized for predicting severity of PCP [4, 5] but with low predictive value. And specific patterns of cytokine response may also have prognostic value as we previously demonstrated that plasma IL-8 and IL-6/IL-10 ratio could be used as biomarker for prediction of prognosis in PCP patients [6]. Interestingly, the secretion of pulmonary cytokines (IL-2, IL-6, IL-17, IL-27, IL-10, IL-35 and IL-22) can be negatively or positively controlled by indoleamine 2, 3-dioxygenase (IDO) [7]. In addition, IDO has been used as a biomarker for predicting occurrence and prognosis of tuberculosis in both HIV-infected patients and community-acquired pneumonia patients [8, 9]. Thus, it is possible that IDO as a biomarker for predicting the severity and/or fatal outcomes of PCP patients.

IDO is an interferon γ–inducible cytosolic enzyme that catabolizes tryptophan (Trp) to kynurenine (Kyn) [10]. Moreover, IDO regulates a broad spectrum of immune responses during chronic infections, the immune-escape of cancer cells, tissue inflammation, transplantation, maternal tolerance toward the foetus and autoimmunity [11]. IDO plays an important role during viral infections including HIV and introduces a bactericidal effector mechanism and also linked to T-cell immunosuppression and tolerance [10]. In addition, it has been reported that high plasma KT ratio was associated with progressive HIV disease and the AIDS dementia complex [12]. Elevated IDO activity was also found in HIV-infected patients with tuberculosis [9]. Moreover, abnormal high KT ratio was also associated with decreased survival in elderly HIV-negative patients [13]. However, the efficacy of the plasma KT ratio used for predicting the severity and clinical outcomes of active PCP in AIDS patients has not been investigated. In this study, we detected the Kyn to Trp ratio to evaluate the value of IDO activity as a predictive biomarker of PCP in AIDS patients.

## Methods

### Study population

All patients (> 18 years of age) diagnosed with AIDS-PCP in the infectious department of The First Affiliated Hospital of Zhejiang University, China from 2014 to 2017 were retrospectively included in the cohort study. A total of 75 patients were included in the cohort study, one was excluded due to bacterial infection, 12 were excluded due to change of antiretroviral therapy (ART), 13 were excluded due to the absence of samples after anti-PCP treatment, eventually 49 were enrolled. We evaluated the historical data within two months of the cohort including the patient’s baseline condition, body mass index, CD4+ cell count, D-dimer, PaO2/FiO2 ratio, albumin, lactate dehydrogenase, C-reactive protein, erythrocyte sedimentation rate, CT scans, kynurenine, tryptophan, treatment, and the time of death. Patients were divided into PaO2/FiO2 > 300 mmHg group (*n* = 31) and PaO2/FiO2 ≤ 300 mmHg group (*n* = 18) at admission; according to anti-PCP treatment, patients were divided into AIDS-PCP diagnosis (PCPdx) group and after treatment of PCP group; according to the clinical outcome, patients were divide into survivors group (*n* = 37) and non-survivors group (*n* = 11).

### Diagnostic criteria

HIV infection was confirmed in accordance with the definitions outlined by the Centre for Disease Control and Prevention. Diagnosis of clinical PCP was based on the subacute onset of an unproductive cough, fever, progressive dyspnoea, and chest discomfort that worsens within days to weeks; arterial partial pressure of oxygen lower than 65 mmHg at time admission; elevated level of lactate dehydrogenase (> 245 U/L); and suggestive radiological findings. Exclusion criteria were as follows: (1) history of administration of agents with activity against PCP (including trimethoprim-sulfamethoxazole (SMZ-TMP), clindamycin, and caspofungin) or glucocorticoid therapy prior to enrollment; (2) evidence of other immune deficiencies (including malignancy, congenital immunodeficiency, and receipt of chemotherapy); and (3) evidence of immune reconstitution inflammatory syndrome (IRIS).

### Acquisition of samples

The first blood samples were obtained when the patients had symptoms and signs and got no antibiotics. The second blood samples were obtained after four weeks of the anti-PCP treatment. A 10 mL sample of peripheral blood was collected using the EDTA-anticoagulant tube. After centrifugation (1500 rpm for 5 min), the supernatant of blood was stored at − 80 °C until it was used.

### Quantification of plasma tryptophan and kynurenine

Plasma samples were thawed from − 80 °C at room temperature. The tryptophan (Trp) and kynurenine (Kyn) concentrations of plasma samples were measured using ultraperformance liquid chromatography–tandem mass spectrometry (UPLC-MS/MS) as previously described [9]. The IDO activity was determined as the ratio of kynurenine to tryptophan and presented in mM/M units.

### Statistical analysis

Discrete data were presented as counts (%), and continuous data were presented as the mean ± standard deviation (SD), unless specified otherwise. The differences between the continuous data were tested using a Mann-Whitney U-test (non-parametric) or Student’s *t*-test (normally distributed). The differences between categorical data were evaluated using a Chi-square test with a Continuity correction or Fisher exact test. To obtain the greatest combination sensitivity and specificity, we used a receiver operating characteristic (ROC) curve to discriminate the IDO activity between AIDS-PCP patients and controls. The Kaplan-Meier method was used to estimate the cumulative survival probabilities. All statistical analyses were performed using SPSS, version 19 (SPSS, Armonk, New York, United States) and considered statistically significant if *p* < 0.05.

## Results

### Clinical characteristics

Table 1 presents the various clinical features of the 49 patients with AIDS-PCP between survivors and non-survivors. None of the patients received PCP prophylaxis prior to admission. Compared with the survivors, non-survivors with AIDS-PCP exhibited a lower albumin and higher D-dimer, CRP, LDH and Kyn/Trp ratio. And we observed that higher mortality in patients with PaO2/FiO2 ≤ 300 mmHg than in those with PaO2/FiO2 > 300 mmHg.

**Table 1:** Clinical characteristics of the AIDS-PCP patients between survivors and non-survivors

	Survivors (*n* = 38)	Non-survivors (*n* = 11)	*P*-value
Sex, M/F	37/1	11/0	NA
Age, yr	40.97 ± 14.29	43.55 ± 16.48	0.684
Body mass index	19.69 ± 2.648	20.90 ± 2.43	0.274
CD4+ cell count, cells/mm3(ul)	59.28 ± 30.38	47.15 ± 28.71	0.387
D-dimer	1314.34 ± 1697.91	5427.91 ± 8050.52	0.010
Albumin	33.05 ± 5.26	25.37 ± 5.99	0.001
CRP	39.88 ± 148.75	91.74 ± 56.27	0.007
ESR	60.23 ± 25.96	66.00 ± 30.17	0.615
P/F ratio, mmHg	398.34 ± 135.17	318.33 ± 106.62	0.144
LDH	339.55 ± 148.75	543.38 ± 328.44	0.045
Kynurenine	2.93 ± 2.19	4.32 ± 3.56	0.051
Tryptophan	15.48 ± 3.13	15.54 ± 3.66	0.952
Kyn/Trp (mM/M)	169.05 ± 72.40	275.91 ± 132.03	0.002
PaO2/FiO2 ≤ 300 mmHg, No. (%)	9 (9/38)	9 (9/11)	0.001
Patients receiving ART, No. (%)	6 (6/38)	4 (4/11)	0.201
Treatment for PCP	38	11	NA

Data are expressed as mean and SD

### IDO as a biomarker for estimation of clinical response in AIDS-PCP patients

**Fig. 1:**
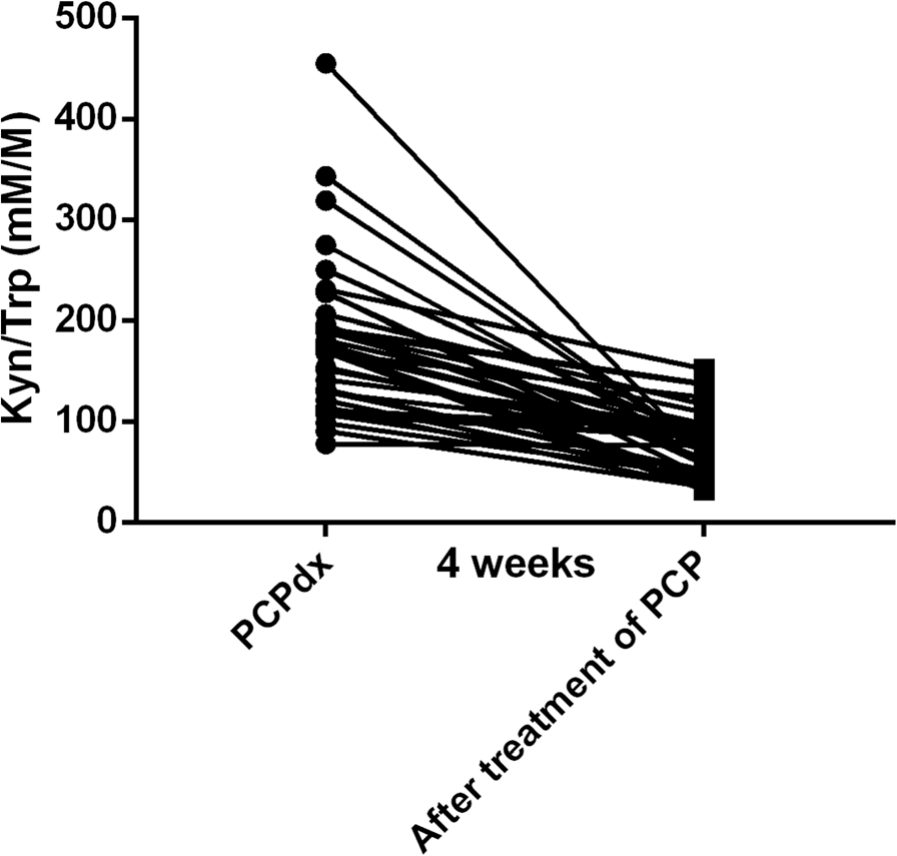
The dynamic of IDO activity of individual patients before and after treatment

The patients treated for four weeks after PCP diagnosis had a significantly lower Kyn/Trp ratio through self-control analysis (175.78 ± 75.80 mM/M vs. 77.20 ± 29.94 mM/M, respectively; *p* < 0.0001). And the changes of Kyn/Trp ratio of individual patients were showed in Fig. 1.

### IDO as a biomarker for prediction of prognosis in AIDS-PCP patients

**Fig. 2:**
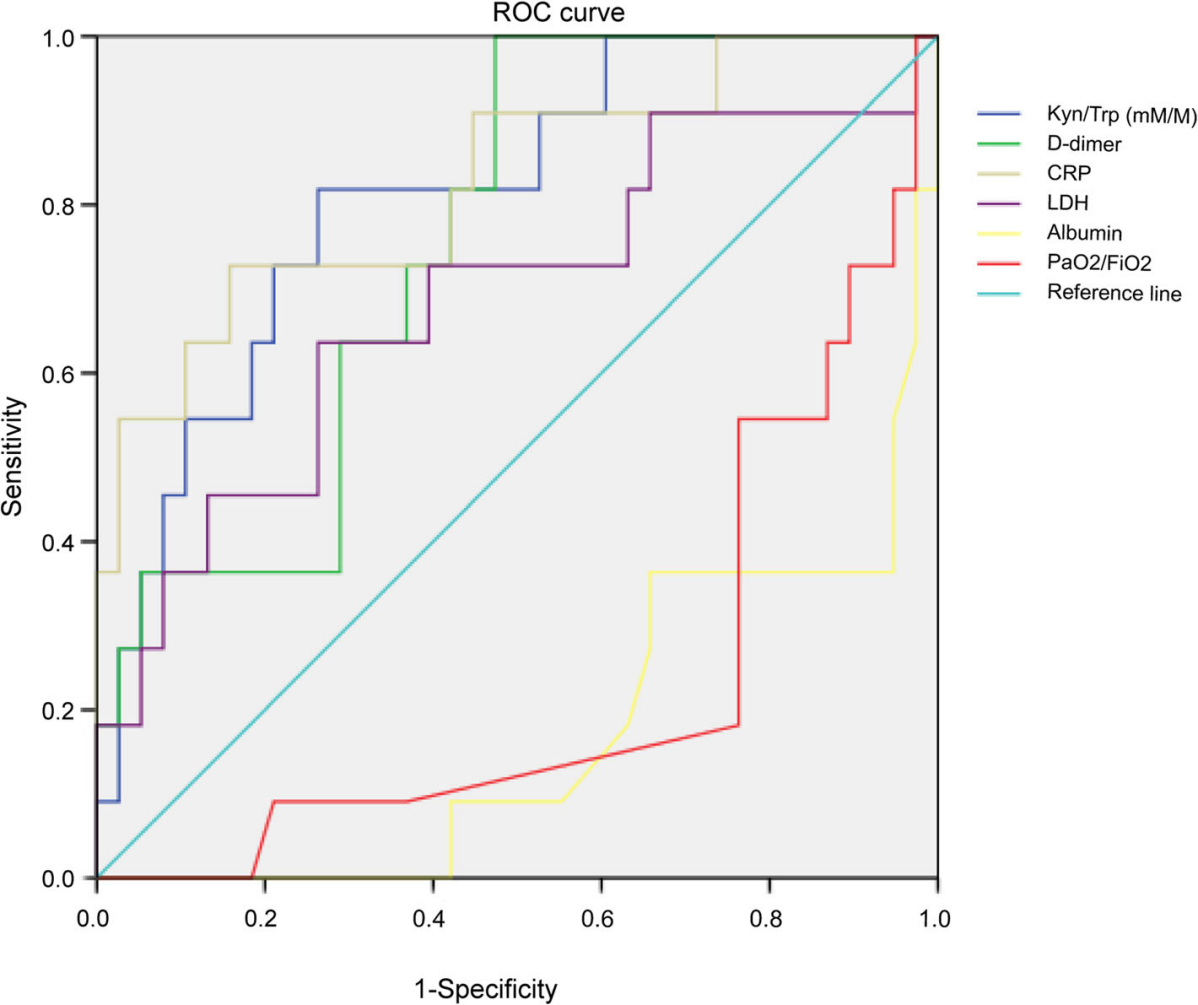
Receiver operating characteristic (ROC) curve analysis of biomarkers in non-survivors (*n* = 11) and survivors (*n* = 38) of AIDS-PCP patients. Kyn/Trp (mM/M) (95%CI 0.673–0.949, *P* = 0.002); AUC =0.811. D-dimer (95%CI 0.612–0.899, *P* = 0.010); AUC = 0.756. CRP (95%CI 0.674–0.977, *P* = 0.001); AUC = 0.825. LDH (95%CI 0.491–0.882, *P* = 0.062); AUC = 0.687. Albumin (95%CI 0.036–0.306, P = 0.001); AUC =0.171. PaO2/FiO2 (95%CI 0.074–0.385, *P* = 0.007); AUC = 0.230

**Fig. 3:**
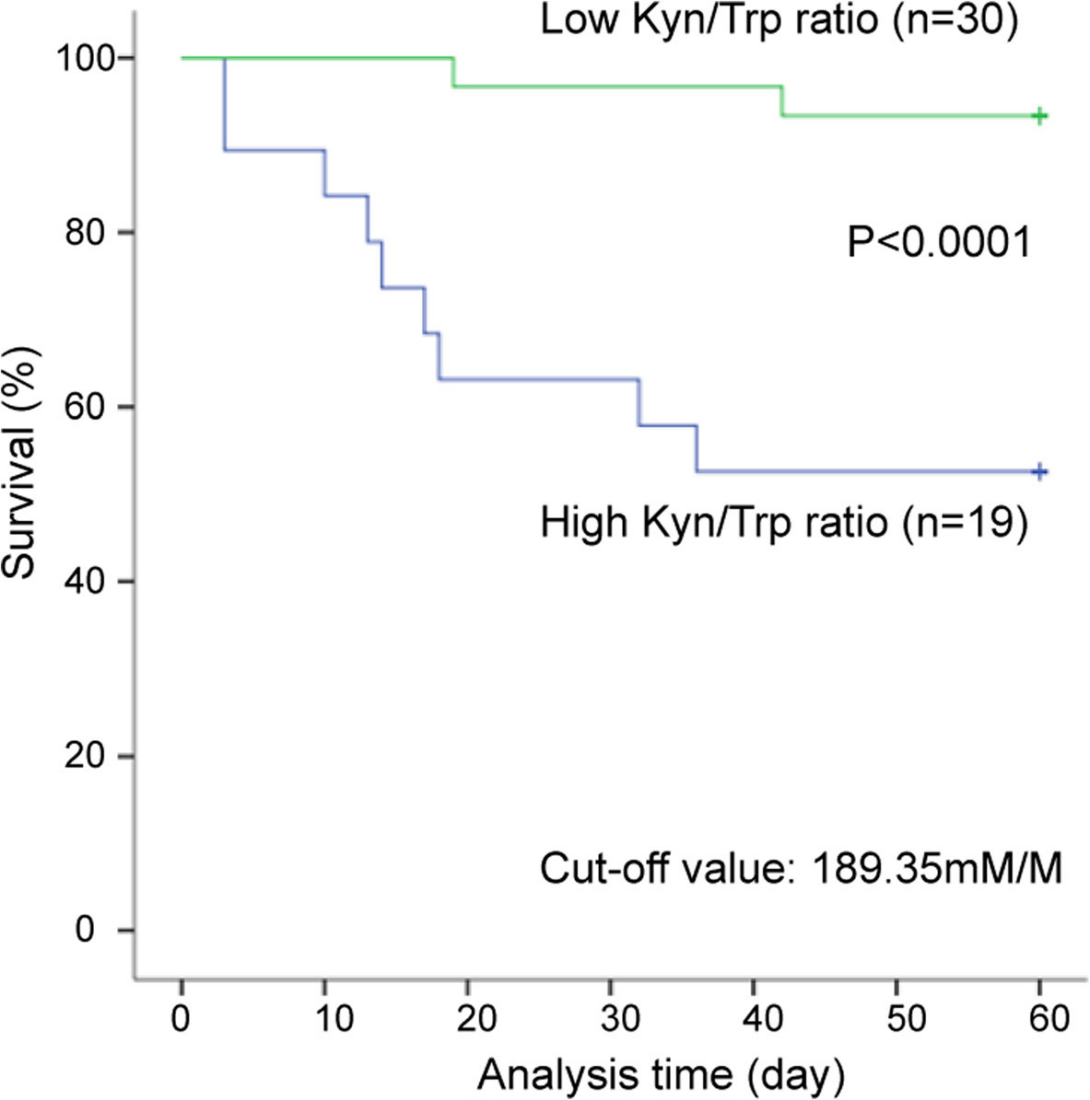
Kaplan Meier curves of AIDS-PCP patients according to different Kyn/Trp ratios

Based on our data, higher Kyn/Trp ratio was observed in the PaO2/FiO2 ≤ 300 mmHg group (*n* = 18), compared with the PaO2/FiO2 > 300 mmHg group (*n* = 31) (241.49 ± 124.56 mM/M vs. 164.91 ± 67.11 mM/M, respectively; *p* = 0.007). And 29 patients (29/31) in the PaO2/FiO2 > 300 mmHg group performed improved conditions, in spite of 2 patients (2/31) were dead within 2 months. However, 9 patients (9/18) were dead within 2 months in the PaO2/FiO2 ≤ 300 mmHg group. Moreover, higher Kyn/Trp ratio was also detected in the non-survivors (*n* = 11), compared with the survivors (*n* = 38) (275.91 ± 132.03 mM/M vs. 169.05 ± 72.40 mM/M, respectively; *p* = 0.002). Furthermore, the ROC curves of Kyn/Trp ratio, D-dimer, CRP, LDH, Albumin and PaO2/FiO2 were listed in Figs. 2. And Kyn/Trp ratio (95%CI 0.673–0.949, *P* = 0.002), D-dimer (95%CI 0.612–0.899, *P* = 0.010) and CRP (95%CI 0.674–0.977, *P* = 0.001) had much higher AUC for predicting death of AIDS-PCP patients. In addition, we obtained an optimal cut-off value (189.35 mM/M) after ROC analysis. Based on the optimal cut-off value, the patients can be divided into high Kyn/Trp group (*n* = 19) and low Kyn/Trp group (*n* = 30). And high Kyn/Trp group had a lower survival rate, compared with low Kyn/Trp group (*P* < 0.0001; Fig. 3).

## Discussion

The present study was the first to measure plasma Kyn and Trp to assess plasma IDO activity in AIDS-PCP patients. We found IDO is a valuable biomarker for estimation of clinical response. And we found that the IDO activity could predict the severity and outcomes of AIDS patients with PCP in a longitudinal HIV-infected cohort study for two months.

IDO was associated with several conditions including cancer, pregnancy and transplantation [14–16]. And the IDO activity in the infectious diseases has also been extensively studied. IDO activity, defined as the serum Kyn/Trp ratio, was found to be significantly higher (2.4-fold) in community-acquired pneumonia(CAP) patients compared with that of the controls [8]. In addition, elevated IDO activity has also been identified in HIV patients expressed as increased levels of serum Kyn but decreased concentrations of serum Trp, and the earlier progression to AIDS [12]. Moreover, there was no significant difference in ART treatment between the two groups in our study although ART may be associated with decreased IDO activity [17, 18].

Currently, there is a lack of available data regarding IDO activity and its clinical significance in fungal diseases, particularly PCP. And precise mechanism of IDO in fungal infectious diseases is not clear. Jiang et al. found that IDO was involved in the immune response to fungal keratitis and was significantly up-regulated in corneal epithelium infected with *Aspergillus fumigatus* (*A. fumigatus*) and human corneal epithelial cells (HCECs) incubated with *A. fumigatus* spores [19]. Moreover, some studies have highlighted the important contribution of IDO in the maintenance of homeostatic in conditions such as Aspergillus infection and allergy [19, 20]. However, other studies suggest that IDO may also dampen protective host immunity, indirectly leading to increased pathogen burden [21]. These differences might be inherent to specific pathogens and/or infection severity. Future studies are required to explore the role of IDO in response to various pathogens.

Our study demonstrated that Kyn/Trp ratio was significantly reduced (*p* < 0.0001) after four weeks anti-PCP treatment. And Clement et al. observed that IDO activity in patients with tuberculosis declined to levels similar to those in controls after 6 months of tuberculosis treatment [9]. However, the mechanism of IDO activity change in clinical response is not clear.

Araujo et al. depicted that the immunological balance mediated by the axis IDO/aryl hydrocarbon receptor is fundamental to controlling the severity of pulmonary paracoccidioidomycosis (PCM) [7]. Moreover, one study revealed that kyn-mediated Trp catabolism can independently predict increased mortality among HIV-infected Ugandan patients [22]. Additionally, some studies have showed the significance of IDO activity in prediction of clinical outcome. In patients with bacteraemia, Huttunen et al. presented that serum IDO activity was significantly higher in non-survivors than in survivors, describing the role of IDO activity as an independent predictor of death in the context of bacteraemia [23]. This was similar to the finding of Tattevin et al., who represented that 43% of septic patients with bacteraemia were associated with higher serum IDO activity and IDO activity was associated with sepsis severity and mortality [24]. Similar to these studies, our data indicate that plasma IDO activity may be a biomarker for prediction of death in AIDS-PCP patients. IDO activity was significantly higher in non-survivors compared to survivors among PCP patients (*P* = 0.002). In addition, we found that PaO2/FiO2 ≤ 300 mmHg group had a higher Kyn/Trp ratio than PaO2/FiO2 > 300 mmHg group of AIDS-PCP patients when they were admitted to the hospital (*p* = 0.007). After two months of observation, we found that 9 (9/18) patients were dead in PaO2/FiO2 ≤ 300 mmHg group and 2 (2/31) patients were dead in PaO2/FiO2 > 300 mmHg group.

However, there were several limitations associated with our study. First, the limited sample size was not enough to determine the significance of IDO activity in AIDS-PCP patients. Second, clinical diagnosis of PCP may have false positive defects. Next, IDO activity in AIDS-PCP patients was presented by measuring their plasma concentrations of Kyn and Trp; however, the specific cell types or tissues were not investigated for the expression of functional IDO in these patients. Third, it remains unclear whether our results can be applied to HIV-uninfected and/or paediatric populations.

## Conclusions

IDO activity was associated with survival of patients with PCP. IDO activity can be one of candidate biomarkers for prediction of prognosis in PCP. Further studies with well-design should be followed to evaluated clinical usefulness of IDO activity in PCP.

## Abbreviations

ART: Antiretroviral therapy
BMI: Body mass index
CRP: C-reactive protein
ESR: Erythrocyte sedimentation rate
IDO: Indoleamine 2,3-dioxygenase
Kyn: Kynurenine
LDH: Lactate dehydrogenase
P/F ratio: PaO2/FiO2 ratio
PCP: Pneumocystis pneumonia
Trp: Tryptophan

## References

[1] Smith CJ, Ryom L, Weber R, Morlat P, Pradier C, Reiss P, Kowalska JD, de Wit S, Law M, el Sadr W (2014). Trends in underlying causes of death in people with HIV from 1999 to 2011 (D:a:D): a multicohort collaboration. Lancet..

[2] Buchacz K, Lau B, Jing Y, Bosch R, Abraham AG, Gill MJ, Silverberg MJ, Goedert JJ, Sterling TR, Althoff KN (2016). Incidence of AIDS-defining opportunistic infections in a multicohort analysis of HIV-infected persons in the United States and Canada, 2000-2010. J Infect Dis.

[3] Fei MW, Sant CA, Kim EJ, Swartzman A, Davis JL, Jarlsberg LG, Huang L (2009). Severity and outcomes of Pneumocystis pneumonia in patients newly diagnosed with HIV infection: an observational cohort study. Scand J Infect Dis.

[4] Sage EK, Noursadeghi M, Evans HE, Parker SJ, Copas AJ, Edwards SG, Miller RF (2010). Prognostic value of C-reactive protein in HIV-infected patients with Pneumocystis jirovecii pneumonia. Int J STD AIDS.

[5] Boulware DR, Hullsiek KH, Puronen CE, Rupert A, Baker JV, French MA, Bohjanen PR, Novak RM, Neaton JD, Sereti I (2011). Higher levels of CRP, D-dimer, IL-6, and hyaluronic acid before initiation of antiretroviral therapy (ART) are associated with increased risk of AIDS or death. J Infect Dis.

[6] Sun J, Su J, Xie Y, Yin MT, Huang Y, Xu L, Zhou Q, Zhu B. Plasma IL-6/IL-10 Ratio and IL-8, LDH, and HBDH Level Predict the Severity and the Risk of Death in AIDS Patients with Pneumocystis Pneumonia. J Immunol Res 2016; 2016:1583951. 10.1155/2016/1583951 PMC4992515 27579328

[7] de Araujo EF, Feriotti C, Galdino NAL, Preite NW, Calich VLG, Loures FV (2017). The IDO-AhR Axis controls Th17/Treg immunity in a pulmonary model of fungal infection. Front Immunol.

[8] Suzuki Y, Suda T, Yokomura K, Suzuki M, Fujie M, Furuhashi K, Hahimoto D, Enomto N, Fujisawa T, Nakamura Y (2011). Serum activity of indoleamine 2,3-dioxygenase predicts prognosis of community-acquired pneumonia. J Inf Secur.

[9] Adu-Gyamfi CG, Snyman T, Hoffmann CJ, Martinson NA, Chaisson RE, George JA, Suchard MS (2017). Plasma Indoleamine 2, 3-dioxygenase, a biomarker for tuberculosis in human immunodeficiency virus-infected patients. Clin Infect Dis.

[10] Schmidt SV, Schultze JL (2014). New insights into IDO biology in bacterial and viral infections. Front Immunol.

[11] Curti A, Trabanelli S, Salvestrini V, Baccarani M, Lemoli RM (2009). The role of indoleamine 2,3-dioxygenase in the induction of immune tolerance: focus on hematology. Blood..

[12] Huengsberg M, Winer JB, Gompels M, Round R, Ross J, Shahmanesh M (1998). Serum kynurenine-to-tryptophan ratio increases with progressive disease in HIV-infected patients. Clin Chem.

[13] Pertovaara M, Raitala A, Lehtimaki T, Karhunen PJ, Oja SS, Jylha M, Hervonen A, Hurme M (2006). Indoleamine 2,3-dioxygenase activity in nonagenarians is markedly increased and predicts mortality. Mech Ageing Dev.

[14] Munn DH, Mellor AL (2007). Indoleamine 2,3-dioxygenase and tumor-induced tolerance. J Clin Invest.

[15] Chang RQ, Li DJ (2017). The role of indoleamine-2,3-dioxygenase in normal and pathological pregnancies.

[16] Ye QX, Xu LH, Shi PJ, Xia T, Fang JP (2017). Indoleamine 2,3-dioxygenase and inducible nitric oxide synthase mediate immune tolerance induced by CTLA4Ig and anti-CD154 hematopoietic stem cell transplantation in a sensitized mouse model. Exp Ther Med.

[17] Zangerle R, Widner B, Quirchmair G, Neurauter G, Sarcletti M, Fuchs D. Effective antiretroviral therapy reduces degradation of tryptophan in patients with HIV-1 infection. Clin Immunol. 2002;104(3):242–7. 10.1006/clim.2002.5231 12217334

[18] Chen J, Shao J, Cai R, Shen Y, Zhang R, Liu L, Qi T, Lu H (2014). Anti-retroviral therapy decreases but does not normalize indoleamine 2,3-dioxygenase activity in HIV-infected patients. PLoS One.

[19] Jiang N, Zhao G, Lin J, Hu L, Che C, Li C, Wang Q, Xu Q, Peng X (2015). Indoleamine 2,3-dioxygenase is involved in the inflammation response of corneal epithelial cells to Aspergillus fumigatus infections. PLoS One.

[20] Romani L, Zelante T, De Luca A, Bozza S, Bonifazi P, Moretti S, D'Angelo C, Giovannini G, Bistoni F, Fallarino F (2009). Indoleamine 2,3-dioxygenase (IDO) in inflammation and allergy to Aspergillus. Med Mycol.

[21] Divanovic S, Sawtell NM, Trompette A, Warning JI, Dias A, Cooper AM, Yap GS, Arditi M, Shimada K, Duhadaway JB (2012). Opposing biological functions of tryptophan catabolizing enzymes during intracellular infection. J Infect Dis.

[22] Byakwaga H, Boum Y, Huang Y, Muzoora C, Kembabazi A, Weiser SD, Bennett J, Cao H, Haberer JE, Deeks SG (2014). The kynurenine pathway of tryptophan catabolism, CD4+ T-cell recovery, and mortality among HIV-infected Ugandans initiating antiretroviral therapy. J Infect Dis.

[23] Huttunen R, Syrjanen J, Aittoniemi J, Oja SS, Raitala A, Laine J, Pertovaara M, Vuento R, Huhtala H, Hurme M (2010). High activity of indoleamine 2,3 dioxygenase enzyme predicts disease severity and case fatality in bacteremic patients. Shock..

[24] Tattevin P, Monnier D, Tribut O, Dulong J, Bescher N, Mourcin F, Uhel F, Le Tulzo Y, Tarte K (2010). Enhanced indoleamine 2,3-dioxygenase activity in patients with severe sepsis and septic shock. J Infect Dis.

